# Iatrogenic Delirium in Patients on Symptom-Triggered Alcohol Withdrawal Protocol: A Case Series

**DOI:** 10.7759/cureus.15373

**Published:** 2021-06-01

**Authors:** Alex Wang, Andrew Park, Ralph Albert, Alyssa Barriga, Leigh Goodrich, Bao-Nhan Nguyen, Erin Knox, Adrian Preda

**Affiliations:** 1 Psychiatry and Neurology, University of California Irvine School of Medicine, Irvine, USA; 2 Psychiatry and Neurology, University of California Irvine Medical Center, Orange, USA; 3 Pharmacy, School of Pharmacy, University of Southern California, Los Angeles, USA; 4 Psychiatry and Neurology, University of California Irvine Health Sciences, Orange, USA

**Keywords:** neuropathology, alcohol withdrawal syndrome, agitation, delirium, benzodiazepine, side effects of medical treatment

## Abstract

In this report, we present a case series involving four patients placed on the Clinical Institute Withdrawal Assessment for Alcohol, Revised (CIWA-Ar) protocol for alcohol or sedative-hypnotic withdrawal syndromes, who developed delirium on sustained or increasing symptom-triggered benzodiazepine dosages. In each of the four cases, delirium was not present on admission and resolved in the hospital itself with fixed benzodiazepine tapers. Cases were selected from an electronic medical record database of patients admitted to a United States-based university hospital and placed on CIWA-Ar between 2017 and 2018. This case series illustrates the major limitations of CIWA-Ar including its subjective nature, its susceptibility to inappropriate patient selection, and its requirement for providers to consider alternative etiologies to alcohol and benzodiazepine withdrawal syndromes. These cases demonstrate the necessity of considering other assessment and treatment options such as objective alcohol withdrawal scales, fixed benzodiazepine tapers, and even antiepileptics. An effective systems-based approach to overcoming these challenges may include setting time limits on CIWA-Ar orders within the electronic health record (EHR) system.

## Introduction

Delirium is a leading cause of acute end-organ dysfunction in hospital settings and occurs in as many as 80% of patients in the intensive care unit (ICU) [1]. Delirium developed in the inpatient setting is associated with severe consequences such as increased mortality, decreased long-term cognitive function, and increased hospital stay [1]. While there are many known etiologies of delirium, all of them lead to a distinct measurable manifestation of brain injury [1]. In cases of alcohol and sedative-hypnotic-related delirium, benzodiazepines have a well-established role in the treatment [2]. However, in cases of non-alcohol and non-sedative-hypnotic-related delirium, benzodiazepines are an independent risk factor for the development of delirium [3-8].

An estimated 40% of hospitalized patients have alcohol dependency with 50% of patients experiencing alcohol withdrawal symptoms when they cut down on or stop drinking [9-11]. Symptoms of alcohol withdrawal typically develop in alcohol-dependent patients within 6-24 hours of their last drink. Minor withdrawal symptoms include insomnia, tremors, anxiety, upset stomach, headache, diaphoresis, hypertension, and tachycardia [12,13]. These symptoms are not life-threatening and generally resolve within one to two days. Major withdrawal symptoms include visual hallucinations, auditory hallucinations, seizures, and delirium; the latter two can be life-threatening [12,13].

In the management of patients suffering from alcohol and sedative-hypnotic withdrawal syndromes, providers are often directed to use standardized instruments such as the Clinical Institute Withdrawal Assessment for Alcohol, Revised (CIWA-Ar). The CIWA-Ar treatment protocol focuses on symptom-triggered therapy where patients are administered benzodiazepines at certain symptom severity thresholds [14]. In comparison to fixed-dosage tapers, symptom-triggered therapy has been shown to decrease total benzodiazepine dosage and decrease treatment duration [15,16].

However, CIWA-Ar has some serious limitations related to its application in the inpatient setting. Because CIWA-Ar relies on patient reporting, it is inappropriate to use it on patients who are nonverbal, those who do not speak English (in the absence of accessible translators), or those in confusional states such as delirium or psychosis. In those situations where CIWA-Ar is not appropriate for treatment of alcohol and sedative-hypnotic withdrawal syndromes, other alternative scales and treatment options need to be considered. Objective alcohol withdrawal scales that rely on vital sign thresholds and physical exam findings may be more useful for treatment in situations where CIWA-Ar cannot be applied [14,17].

We report a case series of four hospitalized adult patients who developed delirium while on CIWA-Ar protocol.

Materials and methods

We describe the treatment course of four admitted patients who were placed on CIWA-Ar protocol for alcohol or sedative-hypnotic withdrawal syndromes. After obtaining approval from the institutional review board, we performed a retrospective chart review of an electronic medical record database of 818 patients admitted to a United States-based university hospital and were started on CIWA-Ar protocol between 2017 and 2018. Of the 818 patients placed on CIWA-Ar, 38 patients were diagnosed with delirium, and four of them met our selection criteria (prevalence: 0.49%). In each of the four cases, the patient developed delirium while on sustained or increasing symptom-triggered benzodiazepine dosages. Significantly, these patients did not exhibit any signs of delirium upon admission, and their symptoms of delirium improved following a fixed benzodiazepine taper.

We defined delirium as an acute organic brain syndrome characterized by a disturbance in attention and awareness and cognition or consciousness that developed over a short period of time and not better explained by another preexisting neurocognitive disorder [18]. This definition of delirium encompasses “acute confusional state” and “encephalopathy” and is based on the American Psychiatric Association’s Diagnostic and Statistical Manual of Mental Disorders, Fifth Edition (DSM-5) guidelines [19].

The four patients were evaluated based on a detailed medical history, physical exam, and laboratory studies that included a urine drug screen and blood alcohol concentration assay. All four patients reported a history of alcohol use disorder, having consumed their last drink within 72 hours prior to their admission. Each patient was placed on CIWA-Ar during their respective hospitalizations. The CIWA-Ar protocol at the hospital involved the application of continuous pulse oximetry, vital sign monitoring every two hours (in the intensive care and cardiac telemetry units) or four hours (in the medical-surgical unit), and nurse scoring of CIWA-Ar every four hours. Depending on the recorded CIWA-Ar score, the nursing staff was directed to administer chlordiazepoxide and lorazepam as needed.

Conversion of benzodiazepine to lorazepam-equivalents for relevant comparisons were based on published equipotent estimates [20]. Furthermore, the administration route was considered and converted appropriately [21-23].

## Case presentation

Table 1 provides a summary of the patient characteristics and risk factors related to delirium during their hospitalization.

**Table 1:** Characteristics of patients and delirium risk factors during hospitalization EtOH: ethyl alcohol; DTs: delirium tremens

Age (years)	Gender	Total lorazepam-equivalents administered (mg)	Delirium risk factors	Comments
47	Male	209	Predisposing: EtOH and benzodiazepine abuse, malnutrition, depression. Precipitating: cardiac arrest, hypothermia, dehydration, EtOH, and benzodiazepine withdrawal. Iatrogenic: intubation, propofol administration, physical restraints	History of multiple past suicide attempts by overdose
57	Male	340.5	Predisposing: EtOH and benzodiazepine abuse, medical comorbidities (congestive heart failure). Precipitating: pneumonia, electrolyte disturbances, EtOH withdrawal. Iatrogenic: intubation, propofol administration, physical restraints, lengthy hospitalization	History of alcohol withdrawal seizures and DTs
52	Female	40	Predisposing: EtOH abuse. Precipitating: subdural hematoma, trauma, hypoxia, EtOH withdrawal. Iatrogenic: intubation, propofol and opioid administration	
67	Male	60.75	Predisposing: EtOH abuse, advanced age, medical comorbidities (hypertension and diabetes). Precipitating: (presumed) vitamin deficiency, trauma, acidosis, EtOH withdrawal. Iatrogenic: physical restraints and opioid administration	Non-English speaking

Patient 1

A 47-year-old male with a past psychiatric history of schizoaffective disorder, post-traumatic stress disorder, major depressive disorder, and alcohol use disorder was brought to a local emergency department (ED) after being found unresponsive outside with bradycardia and subsequent pulselessness. Emergency medical technicians had achieved the return of spontaneous circulation in the field after cardiopulmonary resuscitation and administration of epinephrine. En route to the ED, the patient had become obtunded and was subsequently intubated. Naloxone was administered without response, but subsequent flumazenil elicited spontaneous movement. Laboratory results were notable for a blood alcohol concentration of 368 mg/dL and a urine drug screen was positive for benzodiazepines. On admission, the patient's vitals were significant for hypothermia with a temperature of 92.8 °F. Other admission laboratory studies, including liver function tests, were unremarkable.

**Figure 1:**
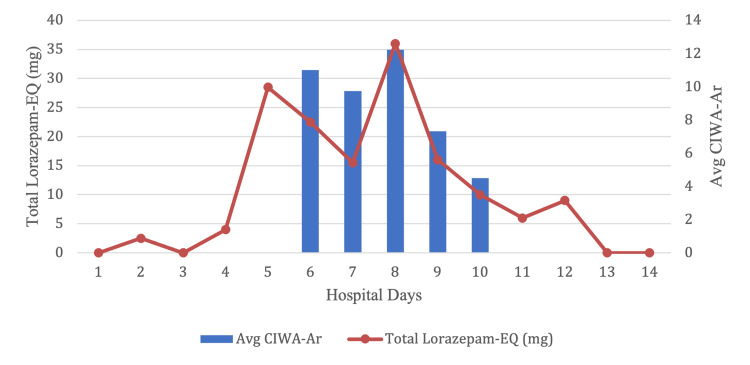
Average CIWA-Ar score in relation to total lorazepam administered CIWA-Ar: Clinical Institute Withdrawal Assessment for Alcohol, Revised

Following ED admission, the patient was admitted to cardiac ICU for post-cardiac arrest care. He was observed to become progressively agitated on hospital day two. Physical restraints were placed, and the patient was sedated with propofol, dexmedetomidine, and midazolam infusions to target Richmond Agitation-Sedation Scale (RASS) scores of 0 to -2. Notably, the patient was administered midazolam 41 mg IV on hospital day five prior to extubation on hospital day six. Given the concern for alcohol and/or benzodiazepine withdrawal, the patient was started on CIWA-Ar. The patient received increasing doses of benzodiazepine, peaking with 36 mg of lorazepam-equivalents on hospital day eight (Figure 1).

Upon clinical exam, the patient was observed to demonstrate waxing and waning of attention, agitation, and orientation only to the self. Serial Confusion Assessment Method for the ICU (CAM-ICU) screens were positive for delirium. On hospital day nine, the patient was started on a fixed dosage of benzodiazepine taper and haloperidol as needed for agitation (Figure 1). Within 24 hours, the patient’s waxing and waning of attention, disorientation, and agitation resolved. Repeated CAM-ICU screens were negative, and the patient was discharged on hospital day 14.

Patient 2

A 57-year-old male with a past psychiatric history of alcohol use disorder and a past medical history significant for congestive heart failure, hepatic steatosis, and past alcohol withdrawal seizures and delirium tremens presented to a local ED with chest pain. He reported consuming up to 2 L of hard liquor per day and stated that he had consumed his last drink on the day of hospital admission. His blood alcohol concentration was found to be 190 mg/dL, and his urine drug screen was positive for benzodiazepines. Liver function tests [aspartate aminotransferase (AST): 129 U/L, alanine aminotransferase (ALT): 58 U/L, alkaline phosphatase (ALP): 160 IU/L, and total bilirubin: 1.5 mg/dL] were suggestive of alcoholic hepatitis. The basic metabolic panel was within normal limits except for mild hypokalemia of 3.1 mmol/L.

Acute coronary syndrome was ruled out. The patient was observed to be encephalopathic on hospital day two, likely due to severe alcohol withdrawal syndrome, and was subsequently intubated and transferred to the medical ICU. He was also started on ceftriaxone and metronidazole due to concerns for aspiration pneumonia. The patient was maintained under sedation on propofol, dexmedetomidine, phenobarbital, midazolam, lorazepam, and chlordiazepoxide, but was observed to be hallucinating and agitated and disoriented with a waxing and waning course following extubation on hospital day 11. The patient screened positive on multiple CAM-ICU assessments. He was physically restrained and administered the scheduled chlordiazepoxide 75 mg oral three times daily, on top of the lorazepam 4 mg IV once and 2 mg IV three times that had been administered over the preceding 24 hours.

He was continued on lorazepam and chlordiazepoxide per the CIWA-Ar protocol, with peak benzodiazepine administration of 52 mg of lorazepam-equivalents on hospital day 13. He was started on a fixed benzodiazepine taper on hospital day 14, and his delirium signs and symptoms slowly improved and resolved by the time of his discharge. The patient’s abnormal laboratory values had normalized and multiple CAM-ICUs administered prior to discharge had been found to be negative.

**Figure 2:**
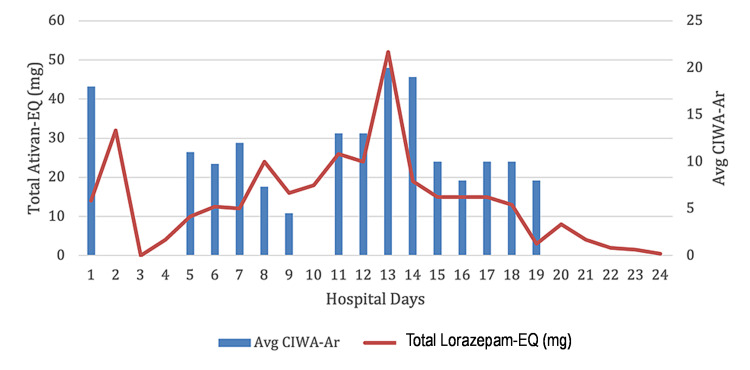
Average CIWA-Ar score in relation to total lorazepam administered CIWA-Ar: Clinical Institute Withdrawal Assessment for Alcohol, Revised

Patient 3

A 52-year-old female was transferred from an outside hospital to a local ED for the management of a subdural hematoma, in light of multiple recent mechanical falls. The patient had a history significant for alcohol use disorder and alcoholic cirrhosis status post-liver transplant five years ago and immunosuppressed on tacrolimus. Her last reported drink was two to three days prior. Upon transfer, the patient was alert and oriented. Her blood alcohol concentration was undetectable, and her urine drug screen was negative. Her other laboratory studies were notable for anemia (hemoglobin: 8.2 g/dL) and thrombocytopenia (platelets: 107,000/μL), with normal coagulation tests.

On hospital day one, the patient experienced a 20-second generalized tonic-clonic seizure, which was aborted with lorazepam. She was subsequently administered a loading dose of levetiracetam and placed on CIWA-Ar protocol given her recent alcohol use. A craniotomy for the evacuation of the subdural hematoma was performed without complications. 

The patient demonstrated progressive fluctuating agitation and disorientation, prompting the administration of lorazepam 13 mg IV on hospital day two and lorazepam 10 mg IV on hospital day three. The patient had a rapid relief team called on hospital day four for the evaluation of acute-onset tachypnea, tachycardia, hypoxemia, and respiratory distress. She underwent rapid sequence intubation, with the administration of propofol, fentanyl, and succinylcholine. Continuous electroencephalogram (EEG) monitoring demonstrated low voltage background and mixed frequencies suggestive of diffuse cerebral dysfunction without epileptiform activity. The patient was taken off of CIWA-Ar and switched to decreasing fixed dosages of chlordiazepoxide. Her agitation and disorientation improved, and she was extubated on the same day. CIWA-Ar scores continued to trend downward until discharge (though only scheduled benzodiazepines were administered, as described above).

**Figure 3:**
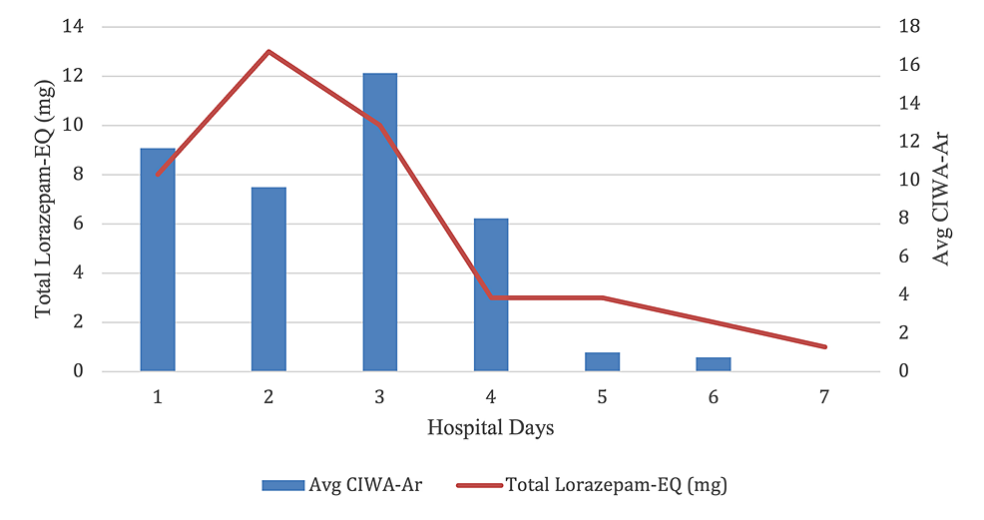
Average CIWA-Ar score in relation to total lorazepam administered CIWA-Ar: Clinical Institute Withdrawal Assessment for Alcohol, Revised

Patient 4

A 67-year-old non-English speaking male was brought to a local ED as a trauma activation after falling from a 15-foot balcony with uncertain loss of consciousness. On arrival to the ED, the patient was alert and oriented. The patient reported consuming his last alcoholic drink on the day of admission. His blood alcohol concentration was 238 mg/dL, and his urine drug screen was negative. Other admission laboratory studies were notable for mild lactic acidosis. No blood thiamine level was assayed during this stay.

The patient denied cardiac symptoms but was admitted to the cardiac telemetry unit for incidentally discovered new-onset atrial fibrillation with a rapid ventricular response. Acute coronary syndrome was ruled out and atrial fibrillation resolved with diltiazem administration. The patient was observed to become progressively agitated on hospital day two. He was subsequently physically restrained and placed on CIWA-Ar protocol. Nursing staff administered lorazepam IV per protocol and morphine as needed. Persistently elevated CIWA-Ar scores led to increasing benzodiazepine administration, peaking with 20 mg of lorazepam on hospital day three.

The patient was observed to demonstrate waxing and waning of attention, orientation, and agitation. He remained on high doses of lorazepam due to elevated CIWA-Ar scores. A brain MRI demonstrated symmetric T2 fluid-attenuated inversion recovery (FLAIR) hyperintensity surrounding the third ventricle and possibly extending to the periaqueductal gray matter and mamillary bodies, a finding commonly seen in the setting of Wernicke's encephalopathy. Physical exam, however, did not reveal any nystagmus, conjugate gaze palsies, pupillary sluggishness, ptosis, anisocoria, or gait ataxia. Chart review did not indicate a history of bariatric surgery nor was there any home diet described. The patient was started on thiamine and switched to a fixed-dosage benzodiazepine taper on hospital day four. The patient’s waxing and waning of attention, orientation, and agitation improved and resolved prior to his discharge.

**Figure 4:**
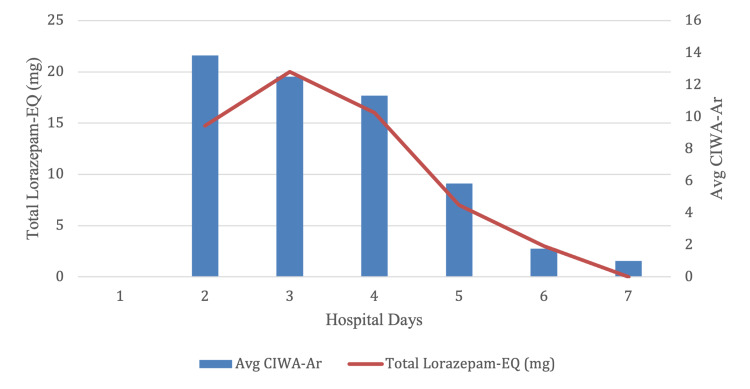
Average CIWA-Ar score in relation to total lorazepam administered CIWA-Ar: Clinical Institute Withdrawal Assessment for Alcohol, Revised

## Discussion

We reported a series of cases in which hospitalized patients developed new-onset or worsening delirium while on the CIWA-Ar protocol. These cases raise concern for the inappropriate use of CIWA-Ar in patients at high risk of delirium that is unrelated to alcohol or sedative-hypnotics. During the patients’ hospital courses, they presented with head trauma, intubation, advanced age, depression, malnutrition, and infections - all of which are known risk factors for delirium [1,24,25]. While initiating CIWA-Ar protocol in these patients was warranted given the unreliable histories provided by them regarding alcohol and benzodiazepine usage, the continuation of the protocol may have been inappropriate given the known risk factors of delirium and the clinical limitations of CIWA-Ar. Benzodiazepines have not been shown to be an effective treatment for non-alcohol-related delirium and have, in fact, been demonstrated to be an independent risk factor in ICU patients [2,6,7,26].

The patients in the cases described experienced worsening delirium symptomatology with escalating benzodiazepine dosages per CIWA-Ar protocol and exhibited rapid resolution of symptoms with fixed benzodiazepine taper. This validates the notion that benzodiazepine overuse was a possible precipitant and likely the perpetuating factor in these patients’ worsening delirium. In case 4, the administration of thiamine for suspected Wernicke’s encephalopathy was started at almost the same time as the fixed benzodiazepine taper, which confounds the assessment of the contribution by benzodiazepines to the patient’s pathology. However, his waxing and waning of attention, orientation, and agitation were described in the absence of key findings consistent with Wernicke’s encephalopathy including ocular abnormalities and cerebellar dysfunction. Although Wernicke’s encephalopathy cannot be ruled out, the patient’s symptoms suggested the presence of delirium.

The CIWA-Ar protocol includes 10 scored criteria: nausea and vomiting, tremor, paroxysmal sweats, anxiety, agitation, tactile disturbances, auditory disturbances, visual disturbances, headache, and orientation and clouded sensorium [26]. Though these parameters were chosen to quantify the severity of withdrawal, many of them are subjective and heavily dependent on patient response or the judgment of the evaluator. In the cases described, many limitations of CIWA-Ar were noted, including subjectivity in scoring, CIWA-Ar administration in inappropriate settings, and assessment-related difficulties caused by language barriers. In cases 1 and 2, symptoms including sweats, agitation, and tremors were attributed to benzodiazepine or alcohol-induced delirium and led to further administration of benzodiazepines as CIWA-Ar scores remained elevated. In both cases, the decision to switch to a fixed benzodiazepine taper led to the resolution of the patients’ delirium.

Another clinical scenario where the CIWA-Ar protocol is limited and potentially dangerous is when patients are obtunded or otherwise limited in their ability to communicate with staff. In case 3, the patient was intubated midway through the hospital stay in response to acute-onset autonomic instability and respiratory distress. While intubated, the patient was unable to communicate with nursing staff who continued to report elevated CIWA-Ar scores based on the inference of subjective scores including anxiety and auditory disturbances. Following the discontinuation of symptom-triggered benzodiazepines, the patient's delirium symptomatology resolved. In case 4, the patient was a non-English speaker. While translation services were readily available at the hospital, the language barrier could still have potentially introduced errors in subjective criteria scores.

## Conclusions

These cases illustrate the necessity for providers to consider employing available assessment and treatment options other than CIWA-Ar, including objective alcohol withdrawal scales, fixed benzodiazepine dosage tapers, and even antiepileptic medications in select patients. Patients with significant risk factors for delirium should be assessed more closely for the possibility of iatrogenic delirium caused or exacerbated by continued symptom-triggered benzodiazepine administration. Modifications to the electronic medical record systems, including setting time limits on CIWA-Ar order sets, can allow for regular reassessments of their appropriate use.
